# Traumatic Hæmatorrhachis

**Published:** 1904-02-20

**Authors:** 


### TRAUMATIC HÆMATORRHACHIS.

Dr. Alexander Miles and Mr. J. C. Carr[1] report an interesting example of this lesion. The patient, æt. 36, a dock labourer, was heaving a heavy sack over his shoulders when he felt a jerk, as of something giving way "right in between the shoulders." He continued his work for an hour afterwards. During the following night he slept well, but next morning was unable to go to work because of pain and stiffness between the shoulders. When he coughed he had a sensation "like a shock of a galvanic battery" from the place in the back where he was hurt, down the spine and legs to the toes. He walked to the hospital, a distance of half a mile, on the morning of the day following the receipt of the injury. On admission it was observed that he kept his dorsal and cervical spine rigid both on attempting to turn round and to stoop. There was tenderness over the upper two or three dorsal spines, but no irregularity of the spinous processes could be discovered. Soon after admission, his legs became numb and he was unable to move them. He could move his arms with difficulty. Respiration was entirely diaphragmatic. Sensation was entirely lost below the level of the third rib at its junction with the sternum and also partially down the inner sides of the arms. There was retention of urine. On the next day there was considerable distension of the abdomen and breathing was becoming embarrassed. On the following day, the fourth after the accident, operation was decided upon, and laminectomy of the seventh cervical and first dorsal vertebræ performed. A large clot was found between the dura mater and the bone, and on its removal there was considerable bleeding from vessels between the dura mater and the bone in front of the cord. The patient died that evening. At the post-mortem examination it was found that the fifth and sixth cervical vertebræ had been separated from one another. The cord and membranes were intact. There had been no fresh bleeding after the operation.

[1] Edin. Med. Jour., Feb. 1904.

